# A commentary on ‘Duloxetine for rehabilitation after total knee arthroplasty: a systematic review and meta-analysis’

**DOI:** 10.1097/JS9.0000000000000618

**Published:** 2023-07-18

**Authors:** Xiumei Tang, Ye Yang, Fuyuan Zheng, Li Ma, Peiyi Li

**Affiliations:** a Department of Respiratory and Critical Care Medicine, Med-X Center for Manufacturing, Frontiers Science Center for Disease-Related Molecular Network, West China Hospital, West China School of Medicine, Sichuan University, Chengdu; b Health Management Center, General Practice Medical Center, West China Hospital, Sichuan University/Institute of Hospital Management, West China Hospital, Sichuan University, Chengdu; c Department of Undergraduate Students, West China School of Medicine, Sichuan University, Chengdu; d Outpatient Department, West China Hospital, Sichuan University/West China School of Nursing, Sichuan University; e Department of Anesthesiology, West China Hospital, Sichuan University, Chengdu; f Laboratory of Anesthesia and Critical Care Medicine, National-Local Joint Engineering Research Centre of Translational Medicine of Anesthesiology, West China Hospital, Sichuan University, Chengdu, P.R. China

Duloxetine is a norepinephrine reuptake inhibitor (SNRI) that inhibits serotonin and norepinephrine reuptake and modulates the descending inhibitory pain pathways in the central nervous system^1^. Duloxetine’s analgesic efficacy in individuals with total knee arthroplasty (TKA), particularly caused by chronic knee osteoarthritis, has been extensively established^2^. We read with great interest the article entitled ‘Duloxetine for rehabilitation after total knee arthroplasty: a systematic review and meta-analysis’. In this study, Yang *et al*.^3^ discussed the efficacy and safety of duloxetine for postoperative recovery after TKA. They concluded that duloxetine reduced pain over a time span of 3 days to 8 weeks, lower cumulative opioid consumption within 1 day, and improved the physical function of the knee with a time span of 1–6 weeks. This paper is undoubtedly impressive in terms of innovative findings but disappointing in its high unexplained heterogeneity, not performing subgroup analysis regarding main outcomes, as well as not discussing the relevance between statistical and clinical differences.

A better understanding of the confounding factors which affect the efficacy and safety of duloxetine is in urgent need^2–5^. Hereby, we presented the following subgroup analysis based on 6 factors: follow-up period, surgery type (knee or hip), the usage of duloxetine (30 or 60 mg), duration of medication administration (<2 weeks or >2 weeks), patients’ mental status at baseline (with and without depression), and patients’ status of central sensitization (with and without central sensitization). We made a comprehensive synthesis of randomized controlled trials investigating the efficacy of duloxetine in both TKA and total hip arthroplasty (THA) and, for the first time, got the raw data from two enrolled trials, which only provided figures in their published articles. We also regrouped the follow-up period according to the guidelines of the Initiative on Methods, Measurement, and Pain Assessment in Clinical Trials (IMMPACT). Pain should be evaluated short-term (≤24 h), medium-term (2–4 weeks), and long-term (3–6 months or longer)^4^.

## Drug variables

Despite several meta-analyses published regarding the efficacy of duloxetine. The optimal dose and duration for duloxetine remain obscure. The dosage of duloxetine should be more than 60 mg when treating neuropathic pain, whereas a third of the studies used 30 mg^2,3^. Our analysis found that both 30 and 60 mg doses of duloxetine were effective in reducing pain. It is astonishing that 30 mg was superior to 60 mg in reducing ambulation pain (30 mg: −0.73 vs. 60 mg: −0.39) and rest pain (30 mg: −0.81 vs. 60 mg: −0.22). Therefore, we would like to suggest a dose of 30 mg daily in patients with total joint arthroplasty.

Moreover, our data also showed that a lengthier administration period of duloxetine was associated with lower pain scores similarly at ambulation (>2 weeks: −0.46 vs. <2 weeks: −0.39) and rest (>2 weeks: −0.50 vs. <2 weeks: −0.13). However, side effects should be closely and cautiously monitored when using duloxetine for more than 2 weeks since most total joint arthroplasties are carried out in day surgery centers.

**Figure 1:**
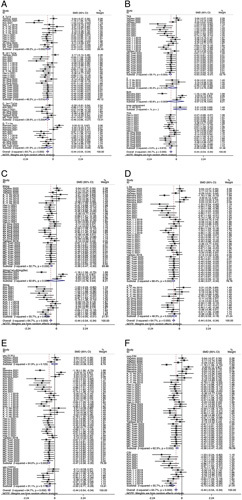
Forest plot of ambulation pain scores (duloxetine vs. placebo).

**Figure 2:**
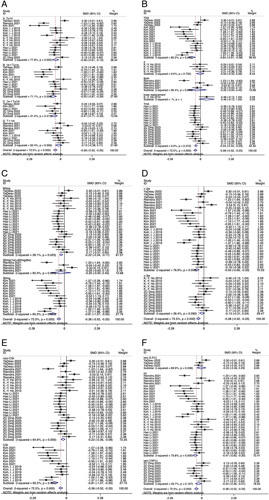
Forest plot of rest pain scores (duloxetine vs. placebo).

In our review, we could not differentiate the effect of duloxetine between TKA and THA patients regarding ambulation (TKA: −0.46 vs. THA: −0.49) and rest (TKA: −0.64 vs. THA: −0.34) pain scores (Figs 1, 2).

## Patient variables

The success of duloxetine administration requires precisely selecting the target population, and identifying the optimization patients is of vital importance. Factors under consideration are patients’ preoperative pain catastrophizing and psychiatric status. In CS patients, the serotonin–norepinephrine descending inhibitory pathway decreases, resulting in a decrease in serotonin and norepinephrine.

Our study found that patients with central sensitization (CS) could benefit more from duloxetine despite the status of measuring pain scores (ambulation: CS −0.73 vs. non-CS −0.36; rest: CS −0.81 vs. non-CS −0.22).

Moreover, it is well-recognized that depression can have a profound influence on pain perception, and the antidepressant effect of duloxetine may upregulate the patient’s pain threshold and emotional experience. Our study reveals that baseline depression status did not influence the effects of duloxetine on pain (ambulation: depression −0.50 vs. non-depression −0.47; rest: depression −0.24 vs. non-depression −0.47).

In conclusion, the current evidence supports the 30 mg and beyond 2 weeks of use of duloxetine for the purpose of reducing postoperative pain. We used appropriate subgroup analysis, which revealed the beneficial patient population and dosing schedule, and dissolving high heterogeneity.

## Ethical approval

Not applicable.

## Consent

Not applicable.

## Sources of funding

Not applicable.

## Author contribution

X.T., Y.Y., and F.Z.: study design and writing; X.T., L.M., and P.L.: language polishing and final approval.

## Conflicts of interest disclosure

The authors declare that they have no conflicts of interest.

## Research registration unique identifying number (UIN)

Not applicable.

## Guarantor

Xiumei Tang and Li Ma.

## Data availability statement

Not applicable.

## Provenance and peer review

Commentary, internally reviewed.

## References

[1] Ding Z Li H Huang C. Significant analgesic benefits of perioperative duloxetine in patients who have depressive symptoms undergoing total hip arthroplasty: a randomized controlled trial. J Arthroplasty 2023;38:519–524. 36252745 10.1016/j.arth.2022.10.007

[2] Azimi A Hooshmand E Mafi AA, Effect of Duloxetine on opioid consumption and pain after total knee and hip arthroplasty: a systematic review and meta-analysis of randomized clinical trials. Pain Med 2023;Apr 7:pnad045. Online ahead of print. 10.1093/pm/pnad045 37027215

[3] Yang JM Wang Y Li JY. Duloxetine for rehabilitation after total knee arthroplasty: a systematic review and meta-analysis. Int J Surg 2023;109:913–924. 37097617 10.1097/JS9.0000000000000230 PMC10389646

[4] Dworkin RH Turk DC Farrar JT. Core outcome measures for chronic pain clinical trials: IMMPACT recommendations. Pain 2005;113:9–19. 15621359 10.1016/j.pain.2004.09.012

[5] Zhong H Li J Chen Y, Effect of duloxetine on pain and opioid consumption after total knee and hip arthroplasty: a systematic review and meta-analysis of randomized controlled trials. Int J Clin Pharm 2023;Jun 9:doi: 10.1007/s11096-023-01593-x. Online ahead of print. 10.1007/s11096-023-01593-x 37294475

